# The End of the Hysterectomy Epidemic and Endometrial Cancer Incidence: What Are the Unintended Consequences of Declining Hysterectomy Rates?

**DOI:** 10.3389/fonc.2016.00089

**Published:** 2016-04-14

**Authors:** Sarah M. Temkin, Lori Minasian, Anne-Michelle Noone

**Affiliations:** ^1^Division of Cancer Prevention, National Cancer Institute, Bethesda, MD, USA; ^2^Division of Cancer Control and Population Sciences, National Cancer Institute, Bethesda, MD, USA

**Keywords:** hysterectomy, corpus uterus, endometrial, cancer

## Abstract

Population-level cancer incidence rates are one measure to estimate the cancer burden. The goal is to provide information on trends to measure progress against cancer at the population level and identify emerging patterns signifying increased risk for additional research and intervention. Endometrial cancer is the most common of the gynecologic malignancies but capturing the incidence of disease among women at risk (i.e., women with a uterus) is challenging and not routinely published. Decreasing rates of hysterectomy increase the number of women at risk for disease, which should be reflected in the denominator of the incidence rate calculation. Furthermore, hysterectomy rates vary within the United States by multiple factors including geographic location, race, and ethnicity. Changing rates of hysterectomy are important to consider when looking at endometrial cancer trends. By correcting for hysterectomy when calculating incidence rates of cancers of the uterine corpus, many of the disparities that have been assumed for this disease are diminished.

## Introduction

Hysterectomy is one of the most frequently performed surgical procedures among women of reproductive age in the United States, second only to cesarean delivery. Approximately 600,000 hysterectomies are performed annually in the United States (1). An estimated 20 million US women have had a hysterectomy; more than one-third of all women have had a hysterectomy by age 60 (1–3). Multiple factors impact hysterectomy rates, including geography and race. Since the 1980s, alternative treatments for menorrhagia, fibroids, and endometriosis have been developed and increased in popularity, leading to decreasing rates of hysterectomy. An inadvertent consequence of these trends toward conservative surgical management of the female genital tract may be an apparent increase in the incidence of gynecologic malignancies specifically cancers of the uterine corpus. Women who have had a hysterectomy are no longer at risk of endometrial or cervical cancer. Failure to remove these women from the population at-risk leads to an underestimate of endometrial cancer incidence rates. Although a higher number of women at risk may lead to additional cases as hysterectomy rates decrease, the incidence rate should not be affected since it is meant to measure the number of new cases per 100,000 women in the population at risk for disease. Gynecologic cancer trends over time also are impacted by changes in the proportion of women with their uterus retained, as they reach ages when these malignancies occur. This paper describes the potential impact of recent changes rates of hysterectomy over time by race and period cohorts.

## Hysterectomy Trends

The majority of hysterectomies are performed for benign indications, with fewer than 15% performed for a malignant preoperative diagnosis (2, 4, 5). The most common primary indications are abnormal uterine bleeding, uterine leiomyomata, and endometriosis (6). Alternatives to hysterectomy including hormonal management, operative hysteroscopy, endometrial ablation, uterine artery embolization, and use of the levo-norgestrel intrauterine device (IUD) as primary management of these conditions have become available and have been demonstrated to be safe (7, 8). The availability of these options has raised questions about potential overuse of hysterectomy. The decreased morbidity associated with uterus-sparing therapies has contributed to their popularity. In addition, the rising age of first pregnancy and improvements in assisted reproduction have made fertility concerns important to women later into life, and contributed to the popularity of uterine preservation. Lastly, although adnexal surgery (e.g., for ovarian cysts) historically triggered a hysterectomy in addition to oophorectomy, the automatic inclusion of hysterectomy in this setting has fallen out of favor. A more conservative approach to the management of ovarian cysts has become more standard, as growing evidence suggests that many ovarian cysts are low risk for malignancy and can safely be monitored by ultrasonography (9, 10). These factors combined have led to recently declining hysterectomy rates (2, 3, 5, 11–13). This decline has been most dramatic among postmenopausal women; the rate of decline has been mostly among white women compared to other racial and ethnic groups (2, 5).

Hysterectomy by the lesser invasive laparoscopic approaches has become more common than either vaginal or abdominal hysterectomy; minimally invasive hysterectomy has also been shown to be an increasingly safer procedure and can be done as an outpatient procedure (3, 4). Population level evidence suggests an increase in all-cause mortality with surgical menopause, resulting in many more women undergoing hysterectomy without oophorectomy (2, 5). Variations in totality of hysterectomy vary by race with black women less likely to have their cervix or ovaries removed with their uterus (14, 15). This trend may be leaving more women undergoing partial procedures in an effort to decrease morbidity and cost, despite the benefits associated with the performance of minimally invasive hysterectomy. Although these changes to patterns of surgical care may affect incidence rates of all gynecologic malignancies, we focus here on the consequences to rates of cancers of the uterine corpus.

## Factors Associated with Hysterectomy

The prevalence of hysterectomy within a population varies by community and patient-level factors. Community-level factors include facility type. It has been observed that the procedure more frequently is performed in community hospitals than academic centers (3). Additional factors, such as physician gender, age, level of education, and local physician density, play a role in whether hysterectomy is recommended and performed (16, 17).

**Figure 1:**
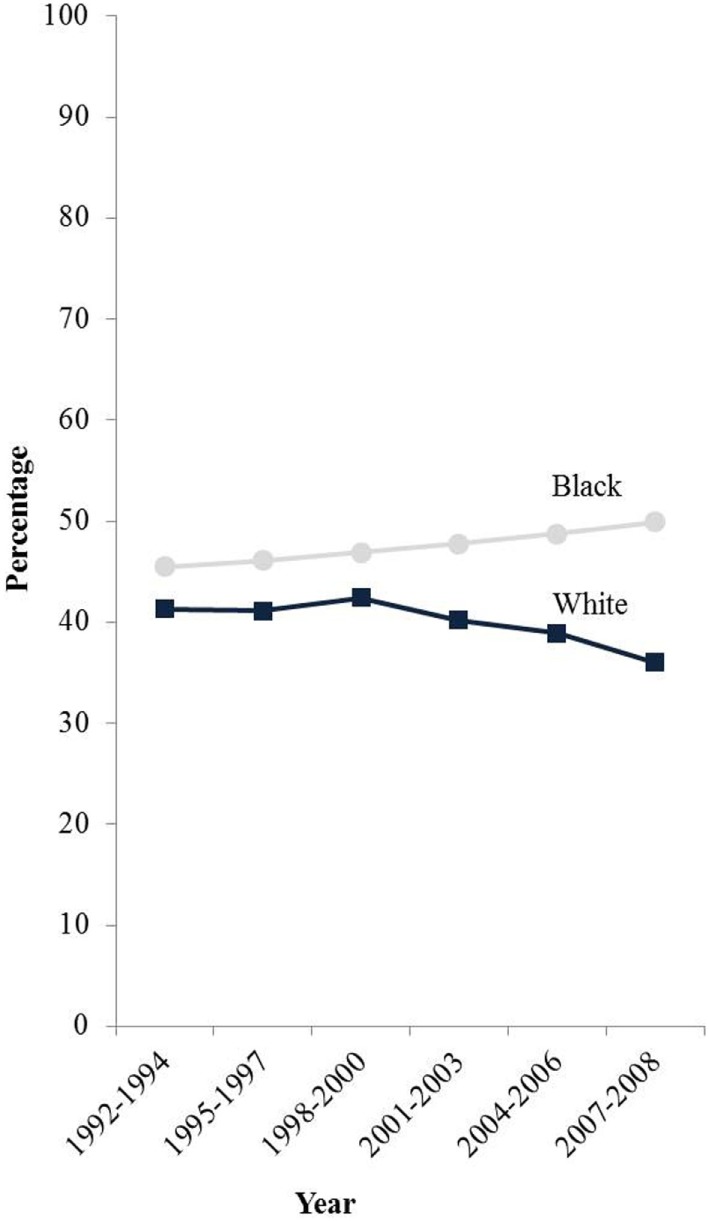
**E-adjusted hysterectomy rates by race among women age 50 and older in the SEER-13 states 1992–2008**. Footnote: data from the Behavioral Risk Factor Surveillance System, Centers for Disease Control and Prevention. States included are: California, Connecticut, Iowa, Georgia, Hawaii, Michigan, New Mexico, Utah, and Washington.

Furthermore, hysterectomy prevalence varies greatly by patient race and ethnicity. Several studies have estimated hysterectomy prevalence from population-based survey data and have shown that hysterectomy rates are markedly higher among black women compared to white and Hispanic women even in recent years (12, 18–20). Specifically, age-adjusted hysterectomy prevalence from 2004 to 2008 in women age 20 and older was 23% among black women compared to 20 and 17% among white and Hispanic women, respectively (18). Hysterectomy prevalence is lowest among Asian and Pacific Islanders (API) (6, 12) and Alaska Native/American Indian women have a prevalence intermediate between black and white women (14, 21). Moreover, hysterectomy prevalence has been declining in the Northeast region of the United States (2). Jamison et al. also showed a decline among white women from the early 1990s to 2008. However, hysterectomy was relatively stable among black women during this time period (Figure 1) (12).

There also are racial and ethnic differences in the prevalence of common benign gynecologic conditions and the use of surgical treatments. Specifically, black women reportedly have higher rates of fibroids, which are the most common benign indication for hysterectomy. Higher rates of hysterectomy are commonly attributed to the greater prevalence of uterine fibroids among black women, rather than a disparity in care. Black women had 3.3 times the odds of receiving a diagnosis of fibroid tumors by pelvic examination, ultrasound scans, or hysterectomy compared to white women in the Nurses’ Health Study II (22). The higher frequency of hysterectomy in the South compared to other geographic regions of the United States is another potential reason that hysterectomy may be more common among black women (3, 18).

Several large studies have reported higher rates of hysterectomy among black women even when adjusted for common clinical and demographic factors that are associated with undergoing hysterectomy (15, 23, 24). An analysis of data from the CARDIA study found black women to have nearly four times the odds of undergoing hysterectomy, compared with white women, after controlling for BMI, polycystic ovarian syndrome, tubal ligation, depressive symptoms, age at menarche, education, access to medical care, geographic site, and a diagnosis of fibroid tumors (OR, 3.7; 95% CI, 2.4–5.6) (23). Similarly, the Study of Women across the Nation (SWAN) included self-reported hysterectomy for benign indications. Black women were 1.7 times more likely to undergo hysterectomy (OR, 1.7; 95% CI, 1.5–1.9) after controlling for education, geographic site, age, marital status, fibroid tumors, parity, smoking, and social support (24). Social determinants of health including differences in patient preferences, physician influence, quality of available care, and access to hysterectomy alternatives also likely influence hysterectomy rates between racial and ethnic groups (15).

## Impact of Hysterectomy on Endometrial Cancer Rates and Trends

Over 60,000 women in the US are expected to be diagnosed with cancers of the uterine corpus in 2001, making it the most common of the gynecologic malignancies (25). Incidence rates for endometrial cancer have continued to climb over the last decade and are projected to continue to increase (26–28). Incidence of uterine cancer has been shown to vary by race and ethnicity, with the highest rates among white women, and the lowest rates among Asian women (29). Incidence rates from the population-based Surveillance Epidemiology and End Results (SEER) program, from 2008 to 2012, were highest among white women (25.8 cases per 100,000 women) followed by black (24.0 cases per 100,000), Hispanic (20.7 cases per 100,000), and Asian Pacific Islander women (19.9 cases per 100,000) (30). Since women who have undergone a hysterectomy are no longer at risk for endometrial cancer, failure to remove them from the ­denominator of the population at risk results in differential underestimation of rates of disease among race and ethnic population (14, 18, 19, 31). Correcting incidence rates by removing these women from the population at risk has been shown to markedly change the rates in the population (18, 19, 21, 31–36). Uncorrected cancer incidence trends over time do not accurately represent the underlying risk of disease, as hysterectomy rates and indications have changed over time and vary by racial groups and geographic region.

The difference in uterine cancer rates between white and black women is diminished after correction for hysterectomy, while the differences between white and Hispanic women are accentuated (12, 18, 19, 31). Specifically, Siegel and colleagues recently reported that hysterectomy-corrected rates among white women in the US were 61% higher, 78% higher for black women, and 47% higher for Hispanic women. Correcting for hysterectomy changed the relative risk of endometrial cancer for black women in the US from 0.87 (95% CI 0.86–0.88) to 0.97 (95% CI 0.94–1.0) making the racial disparity in endometrial cancer incidence between black and white women no longer statistically significant. This underestimation varied greatly by state, which have different rates of hysterectomy. After adjusting for hysterectomy, black women still had a higher risk of uterine corpus cancers in Washington, DC, Florida, North and South Carolina, and a lower risk in Pennsylvania, New Jersey, and New York. The rest of the states with significant disparities lost their significance when corrected for hysterectomy (Alabama, California, Illinois, Indiana, Kentucky, Massachusetts, Michigan, Mississippi, Missouri, Ohio, Tennessee, and Virginia) (18).

**Figure 2:**
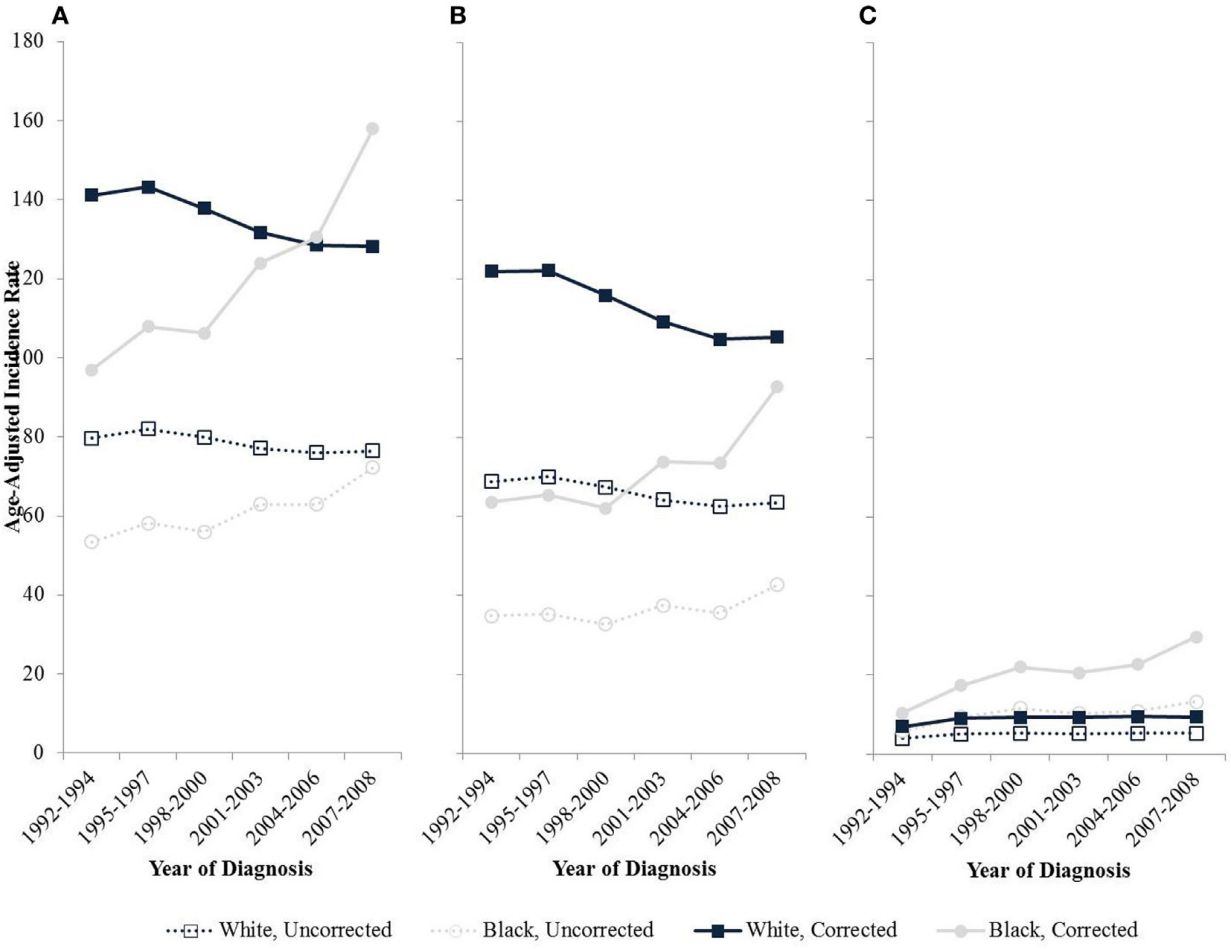
**Age-adjusted endometrial cancer incidence rates by race among women age 50 and older in SEER-13, 1992–2008**. **(A)** All types; **(B)** Type I cancers; **(C)** Type II cancers.

Trends of uterine cancer over time also are distorted since hysterectomy rates are changing over time differentially with respect to race and geography (12). The hysterectomy-corrected incidence of uterine corpus cancers among black women is increasing significantly at 3.1% per year nearly double the 1.8% annual increase based on uncorrected incidence rates (Figure 2). Correction of the incidence trends also reveals a crossover where the incidence for black women is higher than for whites around the mid 2000s bringing the incidence rates of endometrial cancer for black women higher than that of whites (12). The incidence rates for white women have been decreasing since 1992, and the effect is attenuated without correction for hysterectomy. Specifically, incidence rates decrease 0.8% annually after hysterectomy correction compared to an annual decline of 0.5% uncorrected.

A recent analysis from the Epidemiology of Endometrial Cancer Consortium pooled data from seven cohort and four case-control studies and analyzed the effects of known risks for endometrial cancer in white and black women. Obesity, diabetes, smoking, and oral contraceptive use had similar effects on the risk of disease across groups, indicating that the prevalence differences of these risk factors may partially contribute to racial disparities in rates of uterine cancers (37). Adjusting cancer incidence rates corrected for hysterectomy prevalence, particularly when reporting for racial disparities in cancer rates is necessary, given the multiple factors affecting hysterectomy rates including geographic region, race, and ethnicity.

## Risk Factors and Types of Uterine Cancer Over Time

The vast majority of cancers of the uterine corpus arise from the endometrium. Obesity and its associated high circulating estrogen concentrations, is the primary risk factor for the development of endometrial cancer. However, the aging population, the widespread decrease in the use of hormone replacement therapy, particularly progesterone-based agents, population level delays in childbearing, and the increasing prevalence of diabetes all likely factor in the changing incidence over time (26, 27, 38). Endometrial cancer tends to be diagnosed at an early stage with over 80% of the over 55,000 patients with uterine cancer diagnosed with local disease (39). Although the vast majority of women are cured following a diagnosis and intervention of early stage uterine cancer, in 2011, the incidence rate was 27.5 per 100,000 women and the 5-year relative survival rate was 83% for women diagnosed in 2005 to 2011 (39). This is compared to the mid 1970s when the incidence rate was higher at 35.5 per 100,000 women, and the 5-year relative survival was higher at 87% (40). Despite improvements in therapeutic options, 5-year survival appears to have declined (41).

This malignancy has been historically divided into a Type I and Type II based upon the typical biologic behavior of the disease. Type I disease is the more common, low grade form of this malignancy and tends to be diagnosed in younger women and is driven by excess estrogen states such as obesity. The Type I endometrial cancers are usually caught at an early stage where survival is likely. Type II endometrial cancer, including high-grade endometrioid, serous, and clear-cell carcinoma, and carcinosarcomas, however, is typically estrogen independent, occurs in older women and is more likely to be metastatic at diagnosis (42). Increasing proportions of Type II endometrial cancer are being seen in our population and may be due to the aging population where more women age with their uterus intact. This increases the proportion of higher risk and morbid uterine cancers.

Having undergone a hysterectomy for benign indications eliminates the risk for *de novo* development of endometrial cancer. But hysterectomy alternatives are likely altering endometrial cancer risk as well. Consideration can be given for the possibility that women who had a hysterectomy are more likely to have strong risk factors for Type I endometrial cancer (such as those with PCOS, endometriosis, or other hormonal imbalances leading to symptomatic benign gynecologic conditions). Hysterectomy may then selectively remove women at highest risk for low grade malignancies with low mortality rates. Additionally, as women are less likely to undergo hysterectomy and accept alternate therapy for menorrhagia, fibroids, or dysmenorrhea, they may be exposed to hormonal agents such as the levo-norgestrol IUD and other long acting contraceptive agents. These hormonal interventions are protective against Type I endometrial cancer.

After correcting for hysterectomy prevalence, the difference in incidence rates for Type I cancer diminishes between white and black women over time, largely due to the increasing rates of Type I cancers among black women and the decrease in Type I cancers among white women. Much of this difference may be attributable to increased risk factors for endometrial cancer among black women compared to non-black women such as obesity, diabetes, and decreased use of oral contraception (19, 37).

Black women are diagnosed proportionally more frequently with aggressive Type II disease, compared with other racial/ethnic groups (41, 43–49). Uncorrected, incident invasive uterine cancer cases between 1999 and 2006 collected from the Centers for Disease Control and Prevention’s National Program of Cancer Registries or the National Cancer Institute’s SEER Program revealed that only 6.8% of all endometrial cancer patients are black but they represent 17.4% of type II endometrial cancers (48). Rates of Type II cancers appear to be increasing over time entirely due to an increase in incidence among black women (Figure 2) (12). While hormonal hysterectomy alternatives are known be protective against Type I cancers, their role in the development of Type II endometrial cancers is less clear.

The racial disparity in uterine cancer mortality is pronounced. Despite a 30% lower incidence of disease among black women, the mortality rate is 80% higher when compared to whites (49). Histologic differences have historically been used to explain differences in mortality rates between white and black women, and certainly, a larger proportion of Type II malignancies seen in black women contributes to this difference. As with other disparities however, the role of access to care cannot be overlooked. A recent, large analysis of women with Type II endometrial cancer using SEER-Medicare data suggested that controlling for treatment and socioeconomic differences, and medical comorbidities eliminated the difference in the disease-specific mortality between black and white women (50). Disparities related to access to care (specifically hysterectomy alternatives) may amplify the effects of interventions that change risks for the development of gynecologic malignancies. As our understanding of the molecular and genetic factors that correlate to prognosis expands through projects such as the TCGA, ensuring adequate minority participation to clinical trials must be a priority. More research is needed, but many of the disparities in endometrial cancer between black and white women may be explained by hysterectomy rates, access to hormonal hysterectomy alternatives, and differences in risk factors such as obesity.

## Conclusion

An unintended consequence of non-surgical management of common gynecologic conditions appears to be rising incidence and mortality of cancer of the uterine corpus. Incidence and prevalence rates of cancer are useful indicators for assessing the health of a population. Accurate rates are needed in order to determine population level needs and to understand and health disparities among subgroups. As risk reducing surgical removal of other organs (e.g., breast, fallopian tubes, and ovaries) becomes increasingly common, this issue may extend to other cancer disease sites. In endometrial cancer incidence rates uncorrected for hysterectomy have been used to describe wide variations in geographic and racial and ethnic differences in risks of the development of disease. But hysterectomy-corrected rates may help to explain some of the variations as related to patterns of care, access to care, and other non-biologic factors and provide information for appropriately targeting populations to reduce other risk factors such as obesity.

## Author Contributions

Conception: ST and A-MN. Writing: ST, A-MN, and LM. Reviewing: ST, A-MN, and LM.

## Conflict of Interest Statement

The authors declare that the research was conducted in the absence of any commercial or financial relationships that could be construed as a potential conflict of interest. The reviewer BD and handling Editor declared their shared affiliation, and the handling Editor states that the process nevertheless met the standards of a fair and objective review.
